# Laparoscopic peritoneal mucinous cystadenoma debulking: A case report

**DOI:** 10.1097/MD.0000000000041234

**Published:** 2025-01-10

**Authors:** Zhitang Guo, Kui Long, Zhanbin Chen, Wei Zhang, Quanxian Chu

**Affiliations:** a Department of Hepatopancreatobiliary Surgery, The Second Affiliated Hospital of Kunming Medical University, Kunming, China; b Department of General Surgery, Nujiang Prefecture People’s Hospital, Yunnan, Nujiang, China.

**Keywords:** case report, laparoscopy, mucinous cystadenoma, surgery

## Abstract

**Rationale:**

Peritoneal mucinous cystadenoma is rare in the clinic, lacks specific clinical manifestations, tumor markers, and imaging features, and is easily misdiagnosed and missed. Clinical practitioners should maintain a high level of vigilance. Here, we report a case of laparoscopic peritoneal mucinous cystadenoma stripping to improve our understanding of the disease.

**Patient concerns:**

A 34-year-old woman was admitted to our hospital with a history of epigastric pain over the past year that had worsened over the previous 4 months. The patient had no history of trauma or surgery.

**Diagnoses:**

A computed tomography scan of the whole abdomen, as well as hepatobiliary and pancreatic scans and magnetic resonance cholangiopancreatography examinations, showed a low-density mass of approximately 5.8 × 4.8 cm between the right lobe of the liver and the right kidney. The lesion showed no significant enhancement on the enhanced scan, and analysis of tumor markers was normal. The preoperative diagnosis was cholelithiasis with cholecystitis and hepatic cysts.

**Interventions:**

It was proposed to perform “laparoscopic cholecystectomy + hepatic cyst decapitation and decompression” under general anesthesia; however, intraoperative exploration revealed that the abdominal cyst had originated from the right side of the peritoneum and was located between the liver and kidney. The surgical procedure was thus changed to “laparoscopic abdominal cyst removal + cholecystectomy.”

**Outcomes:**

The patient recovered well and was discharged on the fourth postoperative day. Postoperative pathological examination (abdominal cyst) showed mostly serous cells partially covered with high columnar mucus cells, which was consistent with mucinous cystadenoma. The postoperative diagnosis was peritoneal mucinous cystadenoma and cholecystolithiasis with cholecystitis.

**Lessons:**

Clinical diagnosis of mucinous cystadenoma of the abdominal wall is difficult. The possibility of the disease should be considered when a cystic space is found in the abdominal cavity. Diagnosis depends on postoperative pathological examination, and surgery is the preferred treatment option. During the operation, attention should be paid to avoid rupture of the cyst wall and overflow of cyst fluid, and to avoid blind fenestration and drainage or puncture and aspiration sclerotherapy when the diagnosis is unclear.

## 1. Introduction

Mucinous cystadenoma commonly occurs in the ovary, pancreas, and appendix.^[1]^ Mucinous cystadenoma of the appendix is rarely diagnosed preoperatively and may present with various nonspecific signs. Contrast-enhanced computed tomography scans can aid in confirming the diagnosis. Surgical procedures must be conducted with extreme caution to prevent iatrogenic cyst rupture and avoid the spread of mucinous material into the peritoneal cavity.^[2]^ In cases of ovarian tumors, mucinous cystadenomas account for 10% to 15% of all cases and have the potential to develop into pseudomyxoma peritonei (PMP). Treatment for PMP may require repeated surgeries and chemotherapy.^[3]^ Mucinous cystic neoplasm (MCN) of the pancreas is characterized by mucin-producing columnar epithelium and an ovarian-type stroma. MCN typically presents with a slow progression of symptoms, often manifesting as nonspecific gastrointestinal symptoms or abdominal pain. This condition almost exclusively occurs in women aged 40 to 60 years. There is a general consensus that once diagnosed with MCN, timely surgical resection should be performed, regardless of whether the tumor is malignant, due to its potential for malignant transformation.^[4]^ Secondary peritoneal mucinous cystadenoma can occur after rupture of mucinous cystadenoma in other parts; however, its origin from the peritoneum is rare in the clinic. In this case, the tumor originated from the right peritoneum. This report aims to improve our understanding of the disease.

## 2. Case presentation

**Figure 1.**
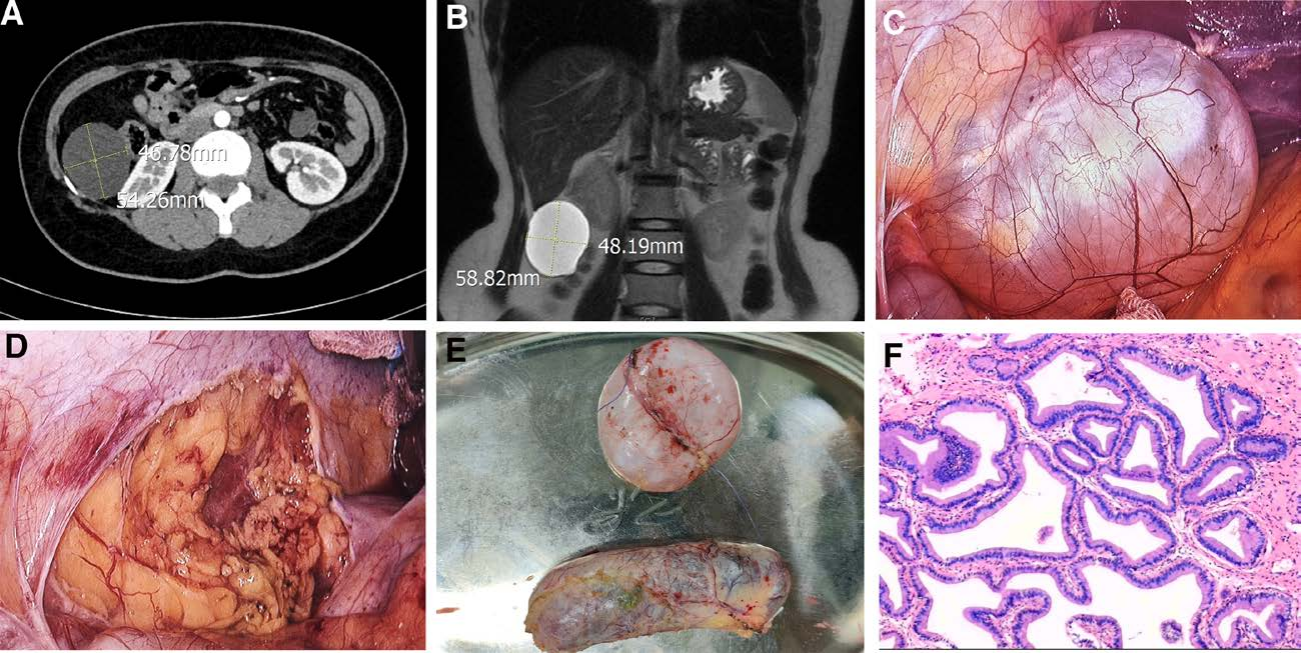
(A and B) The CT and MRI scans revealed a cystic mass measuring 5.8 cm × 4.8 cm. (C) The mucinous cystadenoma of right abdominal wall between liver and kidney. (D) Wound after surgical resection. (E) The excised specimen. (F) Postoperative pathological results. CT = computed tomography.

A 34-year-old woman was admitted to our hospital with a history of epigastric pain over the past year that had worsened over the previous 4 months. The patient presented with intermittent dull pain, without nausea, vomiting, fever, diarrhea, constipation, melena, or mucous stools, and without symptoms of urinary frequency, urgency, or dysuria. At that time, no treatment was initiated due to the mildness of the symptoms. However, 4 months ago, the patient experienced a worsening of symptoms with increased frequency of episodes. There was no history of trauma or surgery, and a computed tomography scan of the whole abdomen, as well as hepatobiliary and pancreatic scans and magnetic resonance cholangiopancreatography examinations, showed a low-density mass of approximately 5.8 × 4.8 cm between the right lobe of the liver and the right kidney. The lesion showed no significant enhancement on the enhanced scan and was considered a hepatic cyst (Fig. 1A and B) and gallbladder stones with cholecystitis. The gallbladder is enlarged, with multiple quasi-circular lesions inside showing high T1 and low T2 signals; the largest lesion has a diameter of approximately 1.1 cm. Layered signals are visible within the cyst, and the cyst wall is mildly thickened. Preoperative laboratory results showed elevated levels of bilirubin, alanine aminotransferase, and aspartate aminotransferase levels were within normal ranges. Renal function, electrolytes, and serum amylase levels were also normal. Analysis of tumor markers indicated that the levels of alpha-fetoprotein, carcinoembryonic antigen, carbohydrate antigen 125, and carbohydrate antigen 199 were normal. The preoperative diagnosis was gallbladder stone with cholecystitis and hepatic cysts. After excluding contraindications to surgery, laparoscopic cholecystectomy and hepatic cyst decapitation and decompression were performed under general anesthesia on May 13, 2024. Intraoperative exploration revealed that the abdominal cyst originated from the right side of the peritoneum and was located between the liver and kidney (Fig. 1C). The surgical procedure was thus changed to “laparoscopic abdominal cyst removal + cholecystectomy.” The peritoneal cyst was completely removed by routine cholecystectomy, with no rupture of the cyst wall or spillage of the cystic fluid (Fig. 1D–E). The surgery was successful, and the patient was discharged on the fourth day after. Surgery the gross specimen showed that the cystic cavity contained a single cyst with smooth grayish-white inner and outer walls and clear cystic fluid. Electron microscopy (abdominal cyst) showed mostly serous cells partially covered with high columnar mucus cells, which was consistent with mucinous cystadenoma (Fig. 1F). The patient experienced no complications postoperatively. During the 5-month follow-up, the patient was in good condition with no signs of recurrence, and we will continue to monitor the patient’s survival.

## 3. Discussion

Peritoneal mucinous cystadenoma is a tumor originating from the glandular epithelial tissue. Continuous secretion of mucus by the glands in adenomas results in the accumulation of secretions in the glandular cavity and gradual expansion. Peritoneal mucinous cystadenomas may show benign, borderline, and malignant characteristics.^[5]^ The pathogenesis of which has not been clarified. It has been suggested to originate from ectopic ovarian tissue,^[6]^ while other studies have proposed that it originates from mucous metaplasia of peritoneal mesothelial cells.^[7]^ Previous studies have also indicated that mutations in the KRAS and GNAS genes are very common in patients with PMP. Multiple studies have demonstrated that these genetic mutations are associated with the occurrence and progression of PMP.^[8]^

It is difficult to diagnose, and most tumors are detected by preoperative physical examination or the presence of clinical compression symptoms, such as abdominal pain and distension. The appearance of clinical compression symptoms is closely related to the location and size of the cyst and requires further clarification by intraoperative exploration and postoperative examination to determine the pathological and histological origin. Mucinous cystadenomas have clear boundaries and present as either single or multiple cysts on the cross-section, and mucus can be seen inside the cyst. Under the microscope, the cyst was seen to be covered with columnar cells, and the presence of an ovarian-like mesenchyme was necessary for the diagnosis of mucinous cystadenoma. A pathological diagnosis is essential for this disease, and surgery is the preferred treatment option.

During surgery, attention should be paid to following the principle of no tumor, protection of the integrity of the cystic cavity, and precise and gentle dissection and separation to avoid rupture of the cyst wall and spillage of the cystic fluid to prevent the occurrence of postoperative implantation and recurrent intestinal obstruction.^[9]^ In addition, several studies have previously confirmed the efficacy of combining hyperthermic intraperitoneal chemotherapy (HIPEC) with cytoreductive surgery (CRS). Hyperthermic intraperitoneal chemotherapy not only effectively eliminates residual tumor cells post-surgery but also significantly reduces the presence of microscopic peritoneal implants, thereby lowering the risk of recurrence.^[10]^

Before surgery, mucinous cystadenoma should be differentiated from serous cystadenoma as well as from hepatic, renal, ovarian, and mesenteric cysts. Differential diagnosis is also necessary for other cystic diseases of the peritoneum or retroperitoneum, such as cystic mesothelioma, also known as benign multicystic peritoneal mesothelioma, which is a rare tumor more commonly seen in middle-aged women and is usually asymptomatic. Given that benign multicystic peritoneal mesothelioma is typically located in the pelvis, it can be challenging to distinguish it from other pelvic and abdominal lesions, such as cystic ovarian masses, particularly mucinous cystadenoma-adenocarcinoma and pseudomyxoma peritonei. Due to its rarity and the lack of specific diagnostic imaging and clinical manifestations, preoperative diagnosis is very difficult, and a definitive diagnosis can only be made through pathological assessment.^[11]^ A detailed history, physical examinations, and ancillary examinations should be performed to improve the accuracy of the diagnosis and to avoid the blind performance of fenestration drainage or puncture aspiration sclerotherapy when the diagnosis is unclear.

## Author contributions

**Conceptualization:** Zhitang Guo, Kui Long, Zhanbin Chen.

**Data curation:** Zhitang Guo.

**Formal analysis:** Zhitang Guo, Kui Long, Zhanbin Chen.

**Funding acquisition:** Zhitang Guo.

**Investigation:** Zhitang Guo, Kui Long.

**Methodology:** Zhitang Guo, Kui Long, Zhanbin Chen.

**Project administration:** Zhitang Guo, Kui Long, Wei Zhang, Quanxian Chu.

**Resources:** Zhitang Guo, Wei Zhang, Quanxian Chu.

**Writing – original draft:** Zhitang Guo.

**Writing – review & editing:** Zhitang Guo, Kui Long.
